# Program Adherence and Effectiveness of a Commercial Nutrition Program: The Metabolic Balance Study

**DOI:** 10.1155/2010/197656

**Published:** 2010-12-21

**Authors:** Cornelia Meffert, Nikolaus Gerdes

**Affiliations:** ^1^Hochrhein-Institute for Rehabilitation Research, Bergseestraße 61, 79713 Bad Saeckingen, Germany; ^2^Department of Quality Management and Social Medicine, Albert Ludwig University Medical Center Freiburg, Engelbergerstr. 21, 79106 Freiburg, Germany

## Abstract

*Objective*. To assess the effectiveness of a commercial nutrition program in improving weight, blood lipids, and health-related quality of life (HRQOL). *Methods*. Prospective observational study with followup after 1, 3, 6, and 12 months with data from questionnaires and blood samples. *Subjects*. After 12 months, we had data from 524 subjects (= 60.6% of the initial samples). 84.1% of the subjects were women. The average BMI at baseline was 30.3 (SD = 5.7). *Results*. After 12 months, the average weight loss was 6.8 kg (SD = 7.1 kg). Program adherence declined over time but was still high after 12 months and showed a positive linear correlation with weight loss. Relevant blood parameters as well as HRQOL improved significantly. *Conclusion*. After 12 months, nearly two thirds of the samples had achieved >5% reduction of their initial weights. The high degree of program adherence is probably due to personal counseling and individually designed nutrition plans provided by the program.

## 1. Introduction

There are numerous studies on effects of therapeutic measures for overweight and obese persons (e.g., [1]). Nevertheless, proof of long-term effectiveness is often not provided [2]. Particularly with regard to commercial diet programs, accurate information about weight loss is rarely available [3]. Many studies can be interpreted and generalized to a limited extent only, either because the size of the sample is too small, the dropout rate is too high, or adherence to the diet is not registered [4]. Numerous studies deal with the—still controversial—issue which form of diet would be optimal for treating the overweight and obese [5–9]. Various studies showed that, in the medium term at least, low-carbohydrate, high-protein diets led to a greater weight loss than low-calorie, low-fat diets [10, 11], though other studies did not yield the same results [12, 13]. Sacks et al. [14] found that the form of the diet had less influence on the success of a weight reduction program than adherence to it and regular contact with the therapist. As short-term therapy plans offer initial success, which is frequently followed by a renewed increase in weight, any therapy aiming at weight loss must meet the criterion of being effective in the long run.

The metabolic balance nutrition program aims at permanently changing the client's lifestyle [15]. Key elements are individualized nutrition plans, drawn up with laboratory support on the basis of the clients' relevant blood parameters. Clients do not receive ready-to-serve meals but individually designed food lists and suggestions to plan meals. Dietary supplements or medications to regulate metabolism are not used. Every client is personally supported by a certified advisor, with the option of either individual or less costly group care. The program does not exclusively address overweight and obese people but also those with normal weight who wish to support a healthy metabolism. It can be considered a low-carbohydrate diet. 

The primary objective of this study was to measure the short-, medium-, and long-term outcome of weight loss achieved during participation as well as the improvement of the relevant blood lipids and health-related quality of life (HRQOL). Program adherence was evaluated at each time point. Particular attention was given to dropout analyses to assess to which extent results can be generalized.

## 2. Material and Methods

### 2.1. Study Design and Assessments

We chose a single-group pre-post observational design as we wished to gain knowledge about the degree of program adherence and the effects of participating in the program under the conditions of a “real-life situation.” That should allow us to generalize the study findings to normal participants in the program. With this study design, we could avoid the more or less artificial study conditions usually associated with randomized controlled trials [16]. The focus of our study thus was on assessing the effectiveness of the program rather than its efficacy.

Participants and advisors filled out questionnaires at a total of five time points: at the start of participation, 4 weeks after the start, and 3 months, 6 months, and one year after. In addition, blood samples were taken at all time points to determine the relevant metabolic parameters. 

Data compiled from clients included, in addition to sociodemographic parameters, baseline data, and psychological factors such as motivation to complete the program and adherence to the program. The latter was assessed by asking about adherence to the eight basic rules of the program [15] (e.g., “Begin every meal with the protein portion”, response categories: “completely,” “mainly,” “sometimes,” “rarely,” “not at all.” The complete set of rules is listed in Table 1). In order to measure HRQOL, we used the “IRES-24 questionnaire” [17], which includes the dimensions “Somatic Health,” “Activities of Daily Living,” “Mental Health,” and “Pain.” The IRES-24 also offers the possibility to compute a sum score of all 24 items. Gender- and age-standardized norms are available for this questionnaire [18].

Advisors were asked to provide information on height and weight of clients and on whether individual or group counseling sessions were attended. To record (co)morbidity of clients we presented a list of 14 illnesses. On this list all relevant illnesses of each client had to be marked. Advisors were furthermore asked to assess the client's motivation on a scale of six (from 1 = very high motivation to 6=not motivated). The advisor questionnaire had to be filled out not only for study participants, but also for clients who refused to participate. We also asked participants who dropped out of the program to fill out a dropout questionnaire to determine reasons for quitting and weight at the time of quitting. 

*N* = 46 advisors took part in the study. In the period from mid-August 2007 to the end of January 2008, each advisor should consecutively include a maximum of 70 clients. Basically all clients who were at least 18 years old and had a sufficient knowledge of German were to be enclosed in the study. The study was reviewed and approved by the ethics committee of the Albert Ludwig University Medical Center Freiburg. The study was explained to the clients, who gave their written informed consent for participation.

### 2.2. Statistical Analyses

We compared clients who refused to participate in the study with study participants, examining the variables age, gender, BMI, motivation, and type of counseling. We used *t*-tests or chi^2^-tests, respectively. At each time point, the same tests were used to determine systematic differences between dropout clients and clients who remained in the study. To compare these groups, we additionally employed the baseline levels of the IRES-24 sum score and the degree to which “individual goals” had been achieved at the previous assessment. The weight change between baseline and 6 months was included in the dropout analyses of the last time point. It was also checked whether the clients included in the study were representative of all clients who began the program in the second half of 2007 (*N* = 30,364). Both groups were compared on the basis of the variables age, gender, and BMI.

Weight changes were analyzed not only per protocol (as treated), but also according to the intention-to-treat principle (ITT), using methods like “last observation carried forward” (LOCF) and “return to baseline” (RTB). To estimate the outcome for nonresponders, we applied a procedure which imputes missing weight data using the expectation maximization algorithm [19]. An “adherence score” was formed from the questions on adherence to the program. For each of the eight basic rules, the response “not at all” was set as “0” while “completely” was set as “4”. This yielded a sum score ranging from “0” (minimum adherence) to “32” (maximum adherence). With respect to changes in the lipid levels, the individual levels of metabolic parameters as well as the ratio of total cholesterol to HDL cholesterol were calculated. The recommendations of the National Cholesterol Education Program (NCEP) [20] and—for the total/HDL cholesterol ratios—of the American Heart Association [21], respectively, served as a basis for interpretations. For the definition of a metabolic syndrome, the NCEP criteria were applied. To interpret the changes in HRQOL, Standardized Response Means (SRMs) and Standardized Effect Sizes (SESs) were calculated. Effect sizes less than 0.5 were considered as small, between 0.5 and 0.8 as medium, and those over 0.8 as large.

Potential predictors of outcome were studied using multiple linear regression analyses. The predictors we chose were initial weight, motivation, type of counseling, the characteristics age, gender, level of education, and marital status, as well as participants' baseline levels of HRQOL. Furthermore, weight changes and adherence to the program were integrated into the regression model.

The alpha error was corrected by Bonferroni adjustment [22]. All analyses were conducted using the Statistical Package for the Social Sciences (SPSS 17.0, SPSS Inc., IL, USA), except for the imputation of missing values, for which the NORM software [23] was applied.

### 2.3. Subjects

The analyses of the treatment results at 12 months were based on the data of *N* = 472 clients. The mean age was 50 years (SD = 12.0) with a range from 19 to 81 years. 84.1% of the clients in the sample were women. 43.0% were employees, 18.2% self-employed, 12.5% housewives, and another 12.3% were retired. 65.5% of the subjects were married. 25.3% suffered from hypertension, 23.9% from muscular-skeletal diseases, and 6.1% from diabetes mellitus. 3.4% had a coronary heart disease, and 3.2% renal insufficiency. Compared with the normative sample, the participants of the study had much poorer baseline measurements on all dimensions of the IRES-24, particularly so on the dimension “mental health.” The weight data were taken mainly from advisors' records (63.2%, *N* = 304). We only fell back on data provided by the clients themselves if advisors' data were missing.

### 2.4. Representativity of the Client Sample

In order to check whether our study clients differed from all the clients who began the program in the second half of 2007, we used data routinely collected for each client in the course of compiling diet plans (*N* = 30,364). With respect to age and gender, no significant differences were found between this sample and the study sample. The BMI at the start of participation showed significant differences (*P* < .001) between the two samples: there were more obese clients in the study population (*M* = 30.3, SD = 6.3) than in the total sample (*M* = 29.2, SD = 5.9). Although significant, the effect size of this difference is quite small (0.18).

## 3. Results

### 3.1. Retention Rates and Dropout Analyses

During the recruitment period, a total of *N* = 970 clients started the program with one of the 46 study advisors. Out of these, *N* = 851 clients gave their informed consent, which amounts to a refusal rate of 14.0% at the start of the study. Analyses of systematic differences showed that clients unwilling to participate in the study were significantly less motivated (*M* = 2.1, SD = 1.0) than study clients (*M* = 1.7, SD = 0.7, *P* < .001), and their BMI (*M* = 28.0,  SD = 5.8) was significantly lower than the BMI of study clients (*M* = 30.2, SD = 6.2, *P* = .001).

If one looks at the retention rates of all clients who consented to participate in the study, one finds a rate of 85.2% at 4 weeks, about ten percent less at 3 months (74.4%), 64.4% at 6 months, and finally a rate of 55.5% at 12 months. At 4 weeks as well as at 3 months, no significant differences were registered between dropouts and clients remaining in the study. At 6 months, however, study clients were significantly more satisfied (*P* < .001) with their “individual goals” achieved (*M* = 6.6, SD = 2.3) than dropout clients (*M* = 5.5, SD = 2.4 on a scale from 0 = no goals achieved to 10 = maximum achievement). The average age of dropouts was also significantly lower (*P* = .005) than that of responders (*M* = 45.7, SD = 12.6 versus *M* = 48.1, SD = 12.1). One year after the start of participation, we recorded similar differences regarding age and the degree of individual achievement of goals. On the parameters of initial weight and weight reduction, however, no significant differences were found.

Almost half of those who dropped out during the study gave as *reason for quitting *that participation in the program was not compatible with the demands of their jobs (49.2%) or their family obligations (40.5%). 29.4% were dissatisfied with the supervision by their advisors (multiple responses were possible). 

As the retention rate of 55.5% was not really satisfactory after 12 months, a follow-up assessment was carried out in order to fill in missing weight data. Thus the rate could be increased to 61.6%.

### 3.2. Program Adherence

Table 1 shows the percentage of clients who answered the corresponding question with “completely” or “mainly.” The category “mainly” can be considered as good program adherence because the program allows occasional “slips” after the first 4 weeks. Throughout the first weeks, however, strict adherence to the rules is required.

Table 1 shows distinctive differences as to how clients adhere to the rules of the program at all time points. At the beginning, it obviously appears to be quite easy to stick to the rules, while it turns out to be rather difficult to adhere to certain rules over a long period of time (e.g., do not eat anything between meals for at least 5 hours). Although program adherence decreased continuously, an average percentage of 68% of all clients followed the rules “completely” or ”mainly” after one year.

### 3.3. Weight Change

The subjects' average BMI of *M* = 30.3  (SD = 5.7) at baseline was reduced to *M* = 27.7  (SD = 4.8) after one year.  Table 2 shows the distribution of the study clients in the various BMI groups at the five time points.

**Figure 1:**
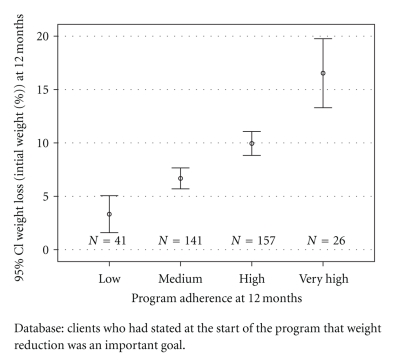
Program adherence and weight loss at 12 months (% of initial weight).

62.5% of the subjects reduced their initial weight by at least five percent at 12 months, and 31.1% lost ten or more percent of their initial weight. Those clients who did not achieve a weight loss of at least five percent had a significantly lower baseline weight (average BMI: *M* = 28.7, SD = 4.8 versus *M* = 31.0, SD = 5.3, *P* < .001). They also had an average adherence score of *M* = 20.4  (SD = 6.3) one year after the start of the program, indicating significantly lower program adherence than the successful clients (*M* = 23.8, SD = 5.3, *P* < .001).  Figure 1 shows the correlation between percentage of weight loss and adherence to the program.

**Figure 2:**
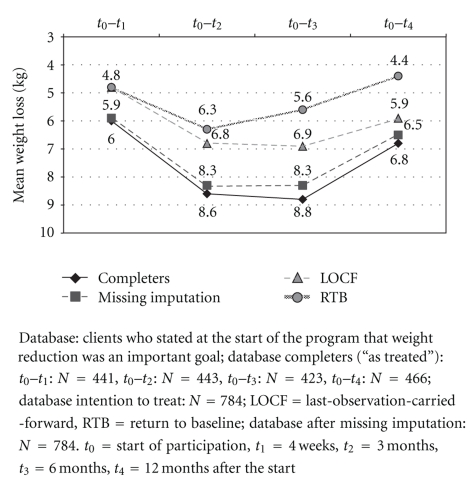
Mean weight loss (kg).

On average, the weight reduction for the subjects who remained in the study at the one-year followup was 6.8 kg (SD = 7.1 kg). Both ITT methods resulted in a lower mean weight reduction at the various follow-up times than the weight reductions reported “as treated” (Figure 2).

### 3.4. Lipids and Metabolic Syndrome

In the long term, the improvement in triglyceride levels was highly significant (*P* < .001). There was also a significant improvement for the total cholesterol and LDL cholesterol levels (*P* = .001 and *P* = .009, resp.). The HDL cholesterol levels also improved during participation in the program. Statistically these changes were not significant, though. The percentage of clients whose ratio of total cholesterol to HDL cholesterol was within the optimal range (<3.5) [21] increased continuously in the short and medium terms. At 12 months, this percentage declined slightly, without however falling back to baseline level. While at the baseline assessment, 14.2% of participants had a metabolic syndrome, this diagnosis applied to only 3.9% of the subjects at 12 months.

### 3.5. Health-Related Quality of Life 

There were significant changes (*P* < .001, resp.) in all dimensions of the IRES-24 and in the sum score at all time points. The treatment effects regarding mental health and somatic health were at the upper end of “medium” effect sizes at the one-year followup. For the sum score, “high” effects were reported. As treatment effects may be influenced by the baseline levels of participants, that is, by the overall available potential for improvement, Table 3 also shows the mean values at the start of participation.

While at baseline, a high percentage of the subjects still had “distinctively” or “severely” poorer levels than the general population, these levels had clearly approached those of the normative sample at the one-year followup.

### 3.6. Predictors of Treatment Results

With respect to successful weight reduction, the degree of adherence to the program and initial weight were strong predictors. Equally important factors were initial weight reduction in the first 4 weeks of participation, and gender (*P* < .01, resp., adjusted *R* ^2^ = 0.463).

## 4. Discussion

A disadvantage of many scientific studies on diet programs is the lack of statements on dropout rates as well as on reasons for quitting, and the fact that results finally reflect only those subjects who remained until the end of the study [25]. In addition, many studies are confronted with the problem of a high “loss to followup” [1]. Especially in nonrandomized studies, analyses of these missing data are an important quality criterion [26]. If no analyses are made of whether the dropouts differ systematically from subjects remaining in the study, it must be assumed that the responders are potentially a selective subgroup and that the results cannot be generalized for all subjects included in the study at baseline. For this reason, dropout analyses were especially important in this study.

During the medium-term and long-term followups, significantly more clients who were younger or dissatisfied with their individual achievement of goals compared with the responders dropped out of the study. With respect to age, gender, and initial BMI, no significant differences were found. Not surprisingly, however, dropouts were less satisfied with their individual goal achievement, even though their mean weight loss at six months was not significantly different. With some reservations then, the results of the study can be generalized to the clients who started the program at baseline.

With respect to age and gender, the sample of the clients included in the study corresponded to all new clients who joined the program in the second half of 2007 (*N* = 30,364). There was a significant difference in initial weight, but the absolute difference was relatively small (1.1 kg, effect size of the difference: 0.18). Therefore it can be assumed that the study clients represent quite well the total of all clients who joined the program during the recruitment period of the study.

According to widely accepted criteria, a weight reduction program is considered successful if a reduction of at least five percent of the baseline weight can be maintained for one year [27]. 62.5% of the study participants achieved this goal. ITT analyses led to a lower average weight reduction at the different follow-up times than the weight losses reported “as treated.” However, both ITT methods are controversial in connection with the evaluation of weight reduction programs [28]. The imputation of missing weight data using the expectation maximization algorithm led to results that closely approach the analyses “as treated”. The question of whether this aspect could make the multiple imputation method the future method of choice for evaluating weight reduction programs cannot be conclusively answered here and should be the subject of further research.

Results from other commercial programs can be compared with findings of this study. A randomized controlled trial (RCT) comparing the Jenny Craig program with a control group reported a mean weight loss of 7.3 kg (SD = 10.4) at 12 months [29]. These data, however, are based on a very small sample (*N* = 32). The same program was the object of a recent effectiveness study including a total of >140 000 clients [30]. After 1 year, a mean weight loss of 13% was registered. As this result, however, is based on the data of only 9% of the clients who enrolled at baseline, it is quite questionable whether these long-term effects can be generalized to all participants in the program, given the dropout rate of >90%. Heshka et al. [31] reported a weight loss of five percent or more for 35% of the subjects after 26 weeks (an RCT comparing the Weight Watchers program to a self-help control group). Overall, it must be stated that independent evaluations of commercial weight reduction programs are rare. Thus, Furlow and Anderson [3] noted correctly, “Numerous commercial programs are available but, unfortunately, accurate information about weight loss with most programs is not available.” In this respect, our study can make a relevant contribution.

Overweight and obesity have a great influence on the HRQOL [32, 33]. International studies show that obesity—in comparison with normal weight—is associated with poorer HRQOL [34]. However, in a meta-analysis Nordmann et al. [28] found that changes in HRQOL are not sufficiently taken into consideration when weight reduction programs are evaluated. In our study, the treatment effects achieved with respect to HRQOL were in the medium to high range. This may be partially due to the poor initial values many participants had in comparison with the normative sample. It is nevertheless surprising that also the dimension “Pain”—which is not necessarily diet-relevant—improved significantly.

In our study, program adherence turned out to be the most important factor for success. We found a linear positive correlation between the degree of program adherence and the outcome. Varying rates of success of dietary programs can probably not be attributed to the type of diet (e.g., low-carb versus low-fat). They are most likely due to the degree of program adherence which particular diets evoke in their participants. Any evaluation of dietary programs would therefore shift the focus from the question “Which type of diet works best to reduce weight?” to the question “Which diet works best to evoke program adherence?”.

As we have no comparative data from other studies, we cannot really determine whether the degree of program adherence found in our study is high or not. We assume, however, that an average of 68% of the participants following the eight basic rules of the program after 1 year within the categories “completely” or “mainly” can be considered as “good program adherence.” We think that this good result was achieved by the “individualization” of the program. Personal nutrition plans on the basis of individual metabolic parameters obviously convey the impression of a diet cut to personal measure, which in turn results in a high identification with the program itself. Personal counseling enhances this identification even more. Thus the diet turns into “my personal nutrition program.”

Compared to other studies about weight reduction programs, we see the strengths of our study in (1) a relatively high retention rate after 12 months (61.6%), (2) meticulously carried out dropout analyses to determine the degree to which the results can be generalised, (3) a comparison of the study sample with all individuals who started the program during the time of recruitment (*N* = 30,364), (4) the inclusion of the outcome parameters lipids and HRQOL, (5) the detailed measurement of adherence to the program, and (6) an assessment of the correlation between program adherence and weight reduction.

### 4.1. Limitations of the Study

The fact that our study is an observational study without a (randomized) control group could be considered a serious limitation. We chose this study design with the intention to measure how the participants reacted to the program in a “real-life situation,” affected by the study itself as little as possible. We also wished to provide data concerning effectiveness which have been missing from the scientific literature despite their importance [30]. One has to bear in mind, however, that an observational study, on principle, can only state covariances between the intervention and the outcomes. It cannot establish a stringent causal relationship between them. Thus, the price to be paid for observing the working of the program in a real life situation seems to be very high. In our study, however, the methodological restrictions of observational studies could be mitigated by the finding of an almost linear positive relationship between adherence to the basic general rules of the program and the central outcome parameters. As we can show that stricter adherence to the program is invariably linked with better outcomes, the conclusion of a causal influence of the program on the outcomes seems legitimate. The “dose-response relation,” which we established in our study, may offer a new approach for observational studies on dietary programs—provided that the “dose,” that is, the degree of program adherence, is measured in detail.

## 5. Conclusions

Participation in the nutrition program led to long-term improvements in health status and HRQOL. The effectiveness of the program has probably to be attributed to the high degree of adherence to the program's basic rules. Comparing various diets, Sacks et al. [14] found that the success of a weight reduction program is not primarily due to a particular type of diet but depends to a great extent on adherence to the program. That corresponds with our results. The main reasons for the high degree of compliance in our study sample appear to be individually designed nutrition plans and personal counseling that bind the clients to “their” nutrition program.

We conclude that program adherence turns out to be a major factor of successful long-term weight reduction, which leads us to recommend a shift of focus. The emphasis of any dietary program should be set on both, the aspect of nutrition as well as the aspect of motivation. One should closely look at the link between motivation and highly individualized weight reduction programs. Psycho-social aspects of compliance will have to be given more consideration in future research.

## Figures and Tables

**Table 1:** Adherence to the eight basic rules of the program.

	4 weeks	3 months	6 months	1 year
Take 3 meals a day	99.0	83.3	75.0	65.4
Do not eat anything between meals for at least 5 hours	96.7	77.3	65.1	52.7
Do not eat anything after 9 p.m.	98.1	94.8	93.6	87.8
Each meal should not exceed 60 minutes	93.5	93.0	91.5	86.5
Begin every meal with the protein portion	98.1	89.0	82.7	68.9
Do not mix different types of protein in one meal	95.9	80.5	69.6	55.1
Take fruit always at the end of the meal	94.5	87.1	77.1	67.8
Drink at least the recommended quantity of water	84.0	72.8	68.6	60.0

Database*: N* = 472*;* data are presented as %, the response categories “completely” and “mainly” were put together.

**Table 2:** BMI groups.

	Total sample (*N*_max_ = 481)					Women (*N*_max_ = 404)					Men (*N*_max_ = 77)				
	*t_0_*	*t_1_*	*t_2_*	*t_3_*	*t_4_*	*t_0_*	*t_1_*	*t_2_*	*t_3_*	*t_4_*	*t_0_*	*t_1_*	*t_2_*	*t_3_*	*t_4_*
Underweight (BMI < 18.5)	0.0	0.0	0.2	0.2	0.0	0.0	0.0	0.3	0.3	0.0	0.0	0.0	0.0	0.0	0.0
Normal range (BMI 18.5 to 24.9)	13.1	24.3	30.7	29.6	27.9	14.6	27.0	34.6	33.5	31.6	5.2	9.9	10.0	7.7	9.1
Preobese (BMI 25.0 to 29.9)	38.3	43.1	45.4	48.9	43.8	37.6	40.0	42.1	45.0	40.4	41.6	59.2	62.9	70.8	61.0
Obese class I (BMI 30.0 to 34.9)	30.1	22.9	17.2	14.7	20.0	28.7	22.2	15.8	14.0	19.3	37.7	26.8	24.3	18.5	23.4
Obese class II (BMI 35.0 to 39.9)	12.5	6.3	4.5	4.5	6.0	12.4	6.8	4.8	4.7	5.9	13.0	4.2	2.9	3.1	6.5
Obese class III (BMI ≥ 40.0)	6.0	3.4	2.0	2.1	2.4	6.7	4.1	2.4	2.5	2.8	2.6	0.0	0.0	0.0	0.0

Database: clients who stated at the start of the program that weight reduction was an important goal; *t*_0_ = start of participation, *t*_1_ = 4 weeks, *t*_2_ = 3 months, *t*_3_ = 6 months, *t*_4_ = 12 months after the start; BMI was categorized according to the World Health Organization's (WHO) recommendations [24]; data are presented as %.

**Table 3:** Health related quality of life (IRES-24-questionnaire).

IRES-dimensions	Baseline values			Effect sizes: SRM (SES)		
	Total sample	Women	Men	Total sample (*N*_max_ = 418)	Women (*N*_max_ = 353)	Men(*N*_max_ = 67)
	Mean (SD)	Mean (SD)	Mean (SD)	*t_0_–t_4_*	*t_0_–t_4_*	*t_0_–t_4_*
Somatic health	6.14 (2.69)	6.13 (2.68)	6.63 (2.26)	0.74 (0.57)	0.72 (0.55)	0.83 (0.69)
Mental health	5.52 (2.07)	5.37 (2.04)	6.42 (2.03)	0.79 (0.75)	0.79 (0.77)	0.80 (0.70)
Activities of daily living	7.21 (2.20)	7.20 (2.24)	7.58 (1.96)	0.55 (0.42)	0.52 (0.40)	0.79 (0.54)
Pain	5.60 (2.54)	5.47 (2.48)	6.40 (2.40)	0.65 (0.54)	0.64 (0.55)	0.69 (0.52)
IRES-24-sum score	6.12 (1.82)	6.04 (1.82)	6.76 (1.53)	0.97 (0.74)	0.95 (0.73)	1.13 (0.88)

Data are presented as standardized response means (SRMs), and standardized effect sizes (SESs). All changes were significant (*P*-values determined by Paired-samples-*t*-tests: *P* < .001). Baseline values range from 0 to 10, 10 representing least restrictions of HRQOL. *t_0_* = start of participation, *t*_4_ = 12 months after the start.
